# Iridescent anterior chamber crystals following minor ocular trauma

**DOI:** 10.4103/0974-620X.53041

**Published:** 2009

**Authors:** Olufunmilola A. Ogun, Stella A. Adegbehingbe

**Affiliations:** Department of Ophthalmology, University College Hospital, Ibadan, Nigeria

**Keywords:** Africans, anterior chamber, iridescent crystals, trauma, uveitis

## Abstract

Anterior chamber crystals occur due to severe ocular trauma or chronic inflammation. The affected eye has often suffered irreversible visual loss. Iridescent crystals are rare and they have interesting clinical features which have been reported commonly among Caucasian populations. This condition has never been reported in an African patient. A 21-year-old Nigerian woman presented with a history of trauma to the left eye and subsequent progressive loss of vision. Polychromatic crystals were observed incidentally in the anterior chamber. This is the first report of this unusual clinical condition in an African patient.

## Introduction

Iridescent crystals are a rare physical sign. They may be found in both the anterior and posterior chambers, but are found more commonly in the latter. Usually, the presence of crystals in the anterior chamber indicates a more serious underlying ocular disorder.[1,2] Iridescent crystals in the anterior chamber appear as tiny refractile deposits within the iris stroma and are best visualized by oblique illumination during slit lamp examination.[3] Existing hypotheses regarding the pathogenesis of iridescent crystals include the belief that they represent abnormally large Russell bodies derived from plasma cells in chronic uveitis,[3] as well as, the notion that they derive from the breakdown of red cell membranes or occur as a result of ocular cholesterolosis.[1] However, the existence of iridescent crystals has not been reliably linked to hypercholesterolemia.[1] Nevertheless, it has been consistently established that eyes with anterior chamber crystals rarely possess significant or useful vision and are more commonly severely inflamed or traumatized.[1,2] Iridescent anterior chamber crystals have been described in patients of the different races across the continents, although this condition has never been reported in an African patient.[1-9] This case is particularly interesting because it resulted from an apparently minor home accident.

## Case Report

A 21-year-old female petty trader presented to the Eye clinic of the University College Hospital, Ibadan, Nigeria, with a two-month history of progressive pain and blurring of vision in the left eye, following an incident of blunt trauma. She had hit her eye against the edge of a door, in the dark. Prior to this incident, she had been completely well and enjoying good vision. There was some pain, tearing, redness, and blurring of vision for which she had resorted to self-medication with an unidentified eye drop. Her vision deteriorated slowly in the following weeks before presentation. Finally, complete loss of vision prompted her for consultation. She denied the use of traditional eye medications and had no known history of sickle cell disease.

**Figure 1:**
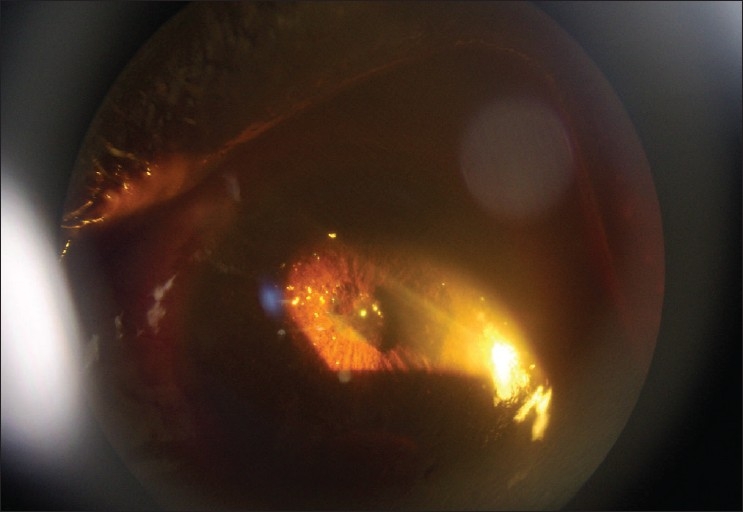
Iridescent anterior chamber crystals, complicated cataract and seclusio pupillae

**Figure 2:**
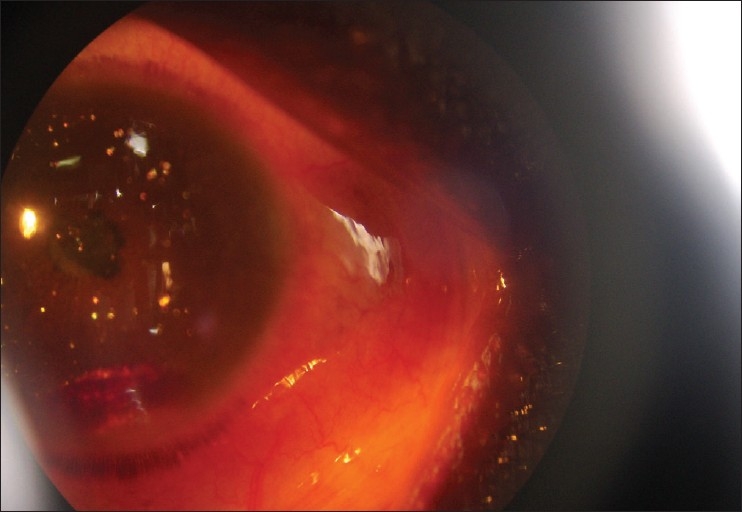
Iridescent crystals and 5 mm layered hyphaema inferiorly

On examination, the right eye was essentially normal. However, in the left eye, a visual acuity of no light perception (NPL) with associated blepharospasm and ciliary injection was observed, though the cornea was clear. The anterior chamber was shallow and contained multiple polychromatic, irregular, flat, crystalloid bodies floating within it, demonstrated by oblique illumination with a narrow slit lamp beam [Figure 1]. Some crystals were embedded superficially on the iris stroma. There was a 5 mm layered hyphaema clot, but no iris neovascularization. The pupil was small, fixed, and irregular and there was a complicated cataract. The degree and exact location of the lens opacity could not be identified due to the seclussio pupillae [Figure 2]. The intraocular pressure was 16 and 19 mm Hg, in the right and left eye, respectively. Anterior chamber flare and cellular activity was difficult to assess due to multiple refractile crystals, but there was no obvious vitreous in the anterior chamber. Visualization of the fundus was obscured by the cataract. The patient was commenced on topical steroids and atropine drops. Ocular ultrasound, haemoglobin electrophoresis (genotype), full blood count, serum urea and electrolytes, and a blood lipid profile were ordered; but the patient defaulted and since then remained lost for follow-up

## Discussion

The presence of crystals in the living eye was first reported by Parfait-Landrau in 1828, followed by Schmidt in 1831. However, Sichel (1850) was the first to identify the anterior chamber crystals as cholesterol.[2]

Cholesterol in the anterior chamber appears as irregularly-shaped multicoloured free-floating crystals, which are larger than cells in size. Anterior chamber cholesterol is believed to be the breakdown product of intraocular blood or blood components derived either primarily from the anterior chamber (as occurs in hyphaema), or as a result of diffusion from the vitreous.[1,2] Crystals may also be embedded in the iris, and suggest a possible history of severe trauma or chronic inflammation. The intraocular pressure is often elevated and vision in such eyes is often irreversibly impaired. Previous trauma or intraocular surgery was found in six out of eight patients in the series by Eagle and Yanoff.^2^ In their review, the average interval between the trauma and the development of iridescent crystals was approximately 13 years.[2]

Our patient had a history of trauma and presented with a painful blind eye after only two months. Neither ocular ultrasound, haemoglobin electrophoresis, microscopy of the anterior chamber crystals, could be done as she did not present herself for investigation. We can only speculate as to the likely pathogenesis of her iridescent crystals. We suppose that she might have developed either a hyphaema or a vitreous haemorrhage or both, from the initial injury with resultant chronic inflammation and secondary cataract formation. The hyphaema may have resolved slowly leaving her with the residual layered clot [Figure 2], and the crystals from the breakdown of heme pigment. On the other hand, if the posterior segment was the primary source of the blood, a subluxation of the lens at the initial injury could have predisposed to vitreous entry into the anterior chamber, which may have carried red blood cells in the event of a vitreous haemorrhage. The crystals were assumed to be cholesterol crystals; any confirmation such as, light microscopic examination, electron microscopy or biochemical tests of the aqueous aspirate, could not be performed due to failure of our patient to follow-up. It would have also been desirable to assess the patient′s genotype, as patients with sickle cell trait and other haemoglobinopathies have a greater tendency to secondary haemorrhage and chronic hyphaema following trauma. This could have predisposed her to the development of chronic posttraumatic uveitis, the posterior synechiae, cataract and iris crystals.[10] Sickle cell trait is highly prevalent in Southwest Nigeria.

Iris crystals have been described frequently in chronic uveitis of which Fuch's iridocyclitis appears to be the most common cause.[3,6-8] Iridescent crystals in the anterior chamber have also been well described in cases of phacolytic glaucoma, in which soluble lens proteins leak into the anterior chamber through an intact capsule.[4,5] The crystals in these cases are a combination of calcium oxalate and cholesterol.[5] Phacolytic glaucoma should be differentiated from phacoanaphylactic (lens-particle induced) glaucoma, in which the lens capsule is disrupted and particles in the anterior chamber are assumed to be of lenticular origin. In contrast to other conditions associated with iridescent crystals, good visual recovery can be obtained in phacolytic glaucoma, following lens extraction.[5] The likelihood that the iridescent crystals, described in this patient, had arisen from the lens; as may occur in phacolytic glaucoma is unlikely. Phacolytic glaucoma is usually associated with a hypermature cataract and significantly raised intraocular pressure, both of which were absent in our patient. The various causes of iridescent crystals in the eye are summarized in Table 1. These include chronic retinal detachment,[9] and hypergammaglobulinaemias.[11]

**Table 1:** Causes of iridescent crystals in the eye including location and composition of crystals

*S.No*.	*Finding (appearance)*	*Condition*	*Composition of crystals*
1	Iridescent crystals free floating in anterior chamber	Cholesterolosis bulbi	Cholesterol
2	Refractile crystals embedded superficially in iris stroma	Chronic uveitis including Fuch's iridocyclitis	Russell Bodies and macrophages
3	Iridescent crystals in aqueous	Phacolytic glaucoma	Calcium oxalate and Cholesterol
4	Lens crystals (intralenticular dust-like refractile particles aligned along sutures)	Myotonic dystrophy	Not true crystals but derivatives of plasma membrane
5	Intralysosomal corneal crystals	Cystinosis	L-cysteine
6	Iris and retina (macular) refractile crystals	Hypergammaglobulinemia	immunoglobulins

In conclusion, the occurrence of iridescent crystals in this patient is interesting, as this will be the first case being described in an African patient and after apparently minor trauma. Possible contributory factors to the development of polychromatic anterior chamber crystals include, neglected ocular trauma, the likelihood of a coexistent sickle cell trait, and failure to seek early appropriate medical attention.
